# Health‐related quality of life around the time of diagnosis in patients with bladder cancer

**DOI:** 10.1111/bju.14804

**Published:** 2019-06-07

**Authors:** Evan Yi‐Wen Yu, Duncan Nekeman, Lucinda J. Billingham, Nicholas D. James, KK Cheng, Richard T. Bryan, Anke Wesselius, Maurice P. Zeegers

**Affiliations:** ^1^ NUTRIM School for Nutrition and Translational Research in Metabolism University of Maastricht Maastricht the Netherlands; ^2^ CAPHRI School for Public Health and Primary Care University of Maastricht Maastricht the Netherlands; ^3^ Department of Public Health, Epidemiology and Biostatistics School of Health and Population Sciences University of Birmingham Birmingham UK; ^4^ MRC Midland Hub for Trials Methodology Research and Cancer Research UK Clinical Trials Unit University of Birmingham Birmingham UK; ^5^ School of Cancer Sciences University of Birmingham Birmingham UK

**Keywords:** quality of life, time of diagnosis, cohort study, QLQ‐C30 questionnaire, #BladderCancer, #blcsm

## Abstract

**Objectives:**

To quantify the health‐related quality of life (HRQoL) of patients with bladder cancer around the time of diagnosis and to test the hypotheses of a two‐factor model for the HRQoL questionnaire QLQ‐C30.

**Methods:**

From participants in the Bladder Cancer Prognoses Programme, a multicentre cohort study, sociodemographic data were collected using semi‐structured face‐to‐face interviews. Answers to the QLQ‐C30 were transformed into a scale from 0 to 100. HRQoL data were analysed in multivariate analyses. The hypothesized two‐factor (Physical and Mental Health) domain structure of the QLQ‐C30 was also tested with confirmatory factor analyses (CFA).

**Results:**

A total of 1160 participants (78%) completed the questionnaire after initial visual diagnosis and before pathological confirmation. Despite non‐muscle‐invasive bladder cancer (NMIBC) being associated with a higher HRQoL than carcinoma invading bladder muscle, only the domain Role Functioning was clinically significantly better in patients with NMIBC. Age, gender, bladder cancer stage and comorbidity all had a significant influence on QLQ‐C30 scores. The CFA showed an overall good fit of the hypothesized two‐factor model.

**Conclusion:**

This study identified a baseline reference value for HRQoL for patients with bladder cancer, which allows better evaluation of any changes in HRQoL as disease progresses or after treatment. In addition, a two‐factor (Physical and Mental Health) model was developed for the QLQ‐C30.

**Abbreviations**
HRQoL: health‐related quality of life
BCPP: Bladder Cancer Prognoses Programme
CFA: confirmatory factor analyses
NMIBC: non‐muscle‐invasive bladder cancer
MIBC: carcinoma invading bladder muscle
EORTC: European Organization for the Research and Treatment of Cancer

## Introduction

Bladder cancer ranks as the 10th most frequently diagnosed cancer worldwide 1, 2. In the UK, the disease accounts for ~10 300 new cases and 5300 deaths per year (Cancer Research UK Cancer Statistics). The majority of patients (75–80%) present with non‐muscle‐invasive bladder cancer (NMIBC) 3. Although not immediately life‐threatening in the majority of cases, recurrence and progression of NMIBC remain significant issues, with up to 55% of patients experiencing recurrence within 5 years of diagnosis 4. Current guidelines therefore recommend long‐term surveillance 5, 6. With the UK prevalence of NMIBC estimated at 46 500 by Cancer Research UK, at any one time there will be at least 40 000 patients requiring surveillance episodes at least once per year.

Typically, surveillance comprises outpatient flexible cystoscopy, with or without urine cytology 7, 8. For patients with low‐risk NMIBC, European Association of Urology guidelines recommend follow‐up cystoscopy and urine cytology at 3 and 12 months after transurethral resection of bladder cancer, and then annually thereafter until 5 years. Patients with high‐risk NMIBC undergo more intensive surveillance: every 3 months for the first 2 years, then every 6 months for the following 3 years, and annually thereafter, most likely for the rest of their lives 9. If recurrence is detected, the tumour is resected and subsequent surveillance will start again, with the frequency determined by the risk category of the recurrence.

Each surveillance cystoscopy and urine cytology episode costs at least £533 10. As a result, bladder cancer is the most costly cancer to treat on a per‐patient basis from diagnosis to death 2, 11. Furthermore, cystoscopy itself significantly increases the burden of disease, as it is an invasive procedure that causes pain and discomfort in about one‐third of patients 12.

As survival amongst patients with NMIBC is high compared with other cancers, and long‐term follow‐up is required, the disease can be considered a chronic disease 13; therefore, health‐related quality of life (HRQoL) plays an important role. To date, however, most HRQoL research has focused on carcinoma invading bladder muscle (MIBC) and, in particular, the differences between different urinary diversions after radical cystectomy 14, 15, 16, but the results are difficult to interpret, as there are often no baseline reference values for comparison. For patients with MIBC, although HRQoL is affected more severely as a result of more radical treatments, a higher chance of metastasis, and worse overall prognosis, it is still an important measure. The few studies that have reported on HRQoL in patients with NMIBC contradict each other 17, 18, 19, 20, 21. In addition to the evidence being sparse, studies have often been hindered by small sample sizes, non‐validated HRQoL questionnaires, and patient populations that are heterogeneous in terms of duration of follow‐up, previous diagnoses of bladder cancer, and number of recurrences or progression.

Additionally, most studies do not take baseline HRQoL into account, which is important for the evaluation of any changes in a patient's HRQoL attributable to disease or treatment. Bladder cancer is a complex disease that often goes undetected for an extended period of time, especially in women 22. This could imply that bladder cancer may have already affected patients’ HRQoL at or around the time of diagnosis, and their HRQoL will not necessarily be the same as that of the general population. It is, therefore, important to study HRQoL at or around the time of bladder cancer diagnosis.

One of the reasons that HRQoL is not often taken into account in studies may result from the complex nature of the questionnaires used to measure HRQoL. More specifically, the commonly used HRQoL questionnaire, the QLQ‐C30 23, has 15 separate domains that need to be compared with each other in order to provide a complete assessment of patients’ HRQoL. Higher‐order models would resolve the issue of too many individual domains. Gundy et al. 24 examined several of these models, including one‐ and two‐factor models, and suggested that a two‐factor model divided into Physical and Mental health provided the best statistical fit.

The aim of the present study was to be the first to quantify and compare HRQoL at or around the time of diagnoses in patients with NMIBC and MIBC separately. Additionally, the study tested the hypothesis of a two‐factor model for the QLQ‐C30.

## Methods

### Study Design

The present study is part of the West Midlands’ Bladder Cancer Prognoses Programme (BCPP), a multicentre cohort study in the West Midlands, UK (ethics reference: 06/MRE04/65; clinicaltrials.gov registration number: NCT00553215). Details of the study have been published previously 25. Briefly, adults (age ≥18 years) presenting at haematuria clinics in nine participating urology centres within the region were enrolled on the basis of abnormal cystoscopic findings suggestive of bladder cancer. Those who had a previous diagnosis of cancer of the urethra, bladder, ureter or renal pelvis within the last decade, HIV infection, or any other condition that might interfere with the safety of the participant were excluded. Enrolment took place between 19 December 2005 and 21 April 2011. All participants provided written informed consent before they were included in the study.

### Data Collection

At the time of diagnoses, semi‐structured face‐to‐face interviews were used by trained research nurses to collect information on socio‐demographics, health‐related lifestyle, medical and drug history, dietary intake, social support and quality of life. The European Organization for the Research and Treatment of Cancer (EORTC) QLQ‐C30 v3 was used for the collection of the HRQoL data. The transformation of the scores is described in detail elsewhere 26. The QLQ‐C30 is a validated questionnaire specifically developed for measuring HRQoL in people with cancer. It has 30 items assessing 15 domains (one global HRQoL domain, five functional domains, and nine symptom domains). Briefly, the answers are converted into a score from 0 to 100, where 100 is the best quality of life and 0 is the worst for all domains except the nine symptom domains, for which 100 is the most problematic symptom and 0 is no symptoms at all. A difference of 10 points or more is considered clinically relevant 27.

The medical records of each patient were reviewed by trained research nurses, and clinicopathological characteristics of bladder cancers at diagnosis were prospectively gathered on dedicated case report forms. This comprised pT stage (according to the TNM 6th edition 2002 classification system 28), grade (according to the WHO 1973 system 29), size of the largest tumour, the number of visible tumours, and comorbidities. Where early re‐resection (within 3 months after first surgery for bladder cancer) indicated an invasive tumour (≥pT2) contrary to the original assessment, then re‐resection pT stage was recorded as the pT stage at diagnosis. Stage was recoded into a numerical outcome variable suitable for analyses by coding pTa tumours with a value ‘1’ and all other stages in sequence. Comorbidities were defined as any pre‐existing medical conditions, or conditions present at the time of bladder cancer diagnosis. For this study, the number of comorbid conditions for each participant was recoded as 0, 1, 2, 3, 4, 5, >5. In addition, a separate variable was generated to recode stage as NMIBC (<pT2) and MIBC (pT2+).

### Statistical Analyses

Mean and sd values were calculated for all domains of the QLQ‐C30 to reflect reference values for patients with NMIBC and those with MIBC. The effect of age, gender, stage and comorbidity on the 15 HRQoL domains from the QLQ‐C30 was analysed using multivariate linear regression models. For this analysis, gender (male, female), stage (pTis, pTa, pT1, pT2, pT3, pT4) and comorbidity (0, 1, 2, 3, 4, 5, >5) were included as categorical variables. Statistical differences between the HRQoL domains and NMIBC and MIBC were tested using the two‐group mean‐comparison (anova). A factor analysis was used to detect the underlying hypothesized two‐factor model, a value of 0.45 was used as the cut‐off value for factor loadings. We used confirmatory factor analyses (CFA) to test our hypothesis of a two‐factor model. The two overall HRQoL items were not included in the CFA, as this domain (overall HRQoL) is not considered to be part of HRQoL but rather a global domain.

## Results

### Sociodemographics

During the enrolment period, patients with symptoms related to bladder cancer accompanied by cystoscopically suspicious lesions were recruited. Out of 1534 recruited participants, 1183 were subsequently diagnosed with bladder cancer. Of these, 1160 had completed the questionnaire before they knew the histologically confirmed diagnosis and could therefore be included in the analyses for the present study.

The majority of the study cohort were men (*n* = 906, 78.1%) and the mean (range) age was 70.4  (26–95) years. Over half of the patients were currently in a relationship (*n* = 728, 62.8%). Most of the patients presented with NMIBC (*n* = 890, 76.7%), with pTa the most common tumour stage (*n* = 575, 49.6%). In addition, 27.4% of the patients had more than five comorbidities, as can be seen in Table 1.

**Table 1:** Socio‐demographic characteristics of study participants.

Demographic	No. of patients	%
Total	1160	100
Men	906	78.1
Women	254	21.9
Age, years		
Mean (sd)	70.4 (10.9)	
Range	26–95	
Marital status		
With partner	728	62.8
Without partner	344	29.7
Missing	88	7.5
Smoking status		
Current	228	19.7
Former	612	52.8
Never	217	18.7
Missing	103	8.8
Tumour stage		
pTis	14	1.2
pTa	575	49.6
pT1	301	25.9
pT2	233	20.1
pT3	1	0.1
pT4	17	1.5
Missing	19	1.6
Tumour grade		
1	254	21.9
2	336	29.0
3	540	46.5
Missing	30	2.6
NMIBC	890	76.7
MIBC	251	21.6
Missing	20	1.7
Comorbidities		
0	92	7.9
1	185	16.0
2	156	13.4
3	176	15.2
4	118	10.2
5	115	9.9
>5	318	27.4

MIBC, carcinoma invading bladder muscle; NMIBC, non‐muscle‐invasive bladder cancer. Missing values are not explicitly reported.

### Health‐Related Quality of Life in Patients with Non‐Muscle Invasive vs Muscle‐Invasive Bladder Cancer

The mean (sd) overall quality of life score was 69 (23) in patients with NMIBC and 61 (24) in those with MIBC. The functional domains for patients with NMIBC ranged from 76 to 86, whilst in patients with MIBC the same domains ranged from 72 to 81. In both groups (NMIBC and MIBC) the worst symptoms were Fatigue and Insomnia. In general, there were few differences in HRQoL between patients with NMIBC and those with MIBC, with none of the scores differing by at least 10 points, except for Role Functioning (Table 2). It was notable that patients with MIBC always scored lower than those with NMIBC on the QLQ‐C30, with a statistically significant difference in seven domains (Global HRQoL, Physical Functioning, Role Functioning, Social Functioning, Fatigue, Pain, Dyspnoea, Insomnia, and Appetite Loss). The lowest scoring domain in both the NMIBC and MIBC groups was Global HRQoL (69/100 and 61/100, respectively). Scores in all other domains were >75 in the NMIBC group and >65 in the MIBC group.

**Table 2:** Health‐related quality of life as measured by the European Organization for Research and Treatment of Cancer questionnaire QLQ‐C30 for non‐muscle‐invasive and muscle‐invasive bladder cancer separately.

QLQ‐C30*	NMIBC (*N* = 890)		MIBC (*N* = 251)		*P*
	Mean	sd	Mean	sd	
Global HRQoL	69	23	61	24	<0.001
Functional domains (0 worst functioning; 100 best functioning)					
Physical functioning	84	20	79	23	0.005
Role functioning	83	28	72	34	<0.001
Emotional functioning	76	22	75	23	0.536
Cognitive functioning	84	19	81	23	0.036
Social functioning	86	23	81	28	0.003
Symptom domains (0 No symptoms; 100 worst symptoms)					
Fatigue	76	23	69	28	<0.001
Nausea and vomiting	96	11	94	13	0.079
Pain	83	25	76	30	<0.001
Dyspnoea	85	24	81	29	0.015
Insomnia	76	31	67	35	<0.001
Appetite loss	89	22	81	31	<0.001
Constipation	88	23	83	28	0.012
Diarrhoea	94	16	93	18	0.471
Financial difficulties	95	17	93	20	0.147

HRQoL, health‐related quality of life; MIBC, carcinoma invading bladder muscle; NMIBC, non‐muscle‐invasive bladder cancer. *P* value is for chi‐squared test. *Score range 0–100.

From Table 3, it is clear that age had a statistically significant effect on nearly all functional HRQoL domains, except for Social Functioning. Additionally, age had a statistically significant effect on Fatigue, Dyspnoea, Insomnia, Constipation, Diarrhoea and Financial Difficulties. Increased age was detrimental in all statistically significant domains except for Financial Difficulties, Insomnia, Diarrhoea and Emotional Functioning. The biggest effect was found in the Physical Functioning domain, where a 10‐year increase in age would decrease the score by 4 points. Women had worse outcomes on every statistically significantly different HRQoL domain compared with men. Gender was the only variable that had a clinical impact (>10 points difference), for instance, on the domain Insomnia where men scored 12.19 points higher. All domains of HRQoL were negatively associated with the number of comorbidities. Patients with NMIBC scored better in every HRQoL domain of QLQ‐C30 compared to those with MIBC, although this was not always a statistically significant difference, nor were any of the differences clinically significant.

**Table 3:** Multivariate linear regression of age, gender, stage and comorbidity on each health‐related quality of life domain.

QLQ‐C30 domains	Age		Gender		Social support		Stage		Comorbidity	
	β	*P*	β	*P*	β	*P*	β	*P*	β	*P*
Global HRQoL	−0.07	0.015	−4.49	0.007	0.30	<0.001	−6.85	<0.001	−2.83	<0.001
Physical F	−0.42	<0.001	−4.83	0.001	0.15	<0.001	−1.77	0.220	−3.21	<0.001
Role F	−0.26	0.002	−1.19	0.577	0.26	<0.001	−8.16	<0.001	−3.26	<0.001
Emotional F	0.28	<0.001	−6.99	<0.001	0.20	<0.001	−0.77	0.640	−1.78	<0.001
Cognitive F	−0.09	0.024	0.61	0.685	0.19	<0.001	−1.78	0.236	−1.82	<0.001
Social F	0.01	0.836	−2.87	0.113	0.22	<0.001	−4.03	0.026	−2.24	<0.001
Fatigue	−0.12	0.029	−5.26	0.002	0.26	<0.001	−5.02	0.004	−3.61	<0.001
Nausea and vomiting	0.06	0.072	−5.33	<0.001	0.04	0.027	−1.23	0.166	−0.60	0.002
Pain	−0.02	0.824	−4.01	0.037	0.25	<0.001	−5.43	0.005	−3.56	<0.001
Dyspnoea	−0.21	0.004	0.06	0.972	0.09	0.021	−2.64	0.153	−3.24	<0.001
Insomnia	0.37	<0.001	−12.19	<0.001	0.25	<0.001	−8.41	<0.001	−3.22	<0.001
Appetite loss	−0.02	0.771	−7.81	<0.001	0.25	<0.001	−7.06	<0.001	−1.15	0.003
Constipation	−0.30	<0.001	−2.50	0.171	0.13	0.002	−3.02	0.098	−1.28	0.001
Diarrhoea	0.11	0.020	−2.72	0.029	0.11	<0.001	−0.67	0.592	−1.14	<0.001
Financial difficulties	0.24	<0.001	0.07	0.958	0.20	0.03	−2.17	0.097	−1.05	<0.001

HRQoL, Health‐related quality of life. β, unstandardized coefficient of the multivariate linear regression analyses; F represents a functional domain.

### Confirmatory Factor Analyses of the Two‐Factor Model

The factor analyses showed that eight items (items 11, 13, 14, 15, 16, 17, 25 and 28) did not fit the two‐factor model (factor loading <0.45), most of which pertained to the one‐item domains such as Sleep (item 11), Appetite Loss (item 13), Constipation (item 16), Diarrhoea (item 17), and Financial Difficulties (item 28), as well as the two‐item domains such as, Nausea and Vomiting (items 14 and 15), and Cognitive Functioning (items 20 and 25). The domains Fatigue (items 10, 12, 18), Physical Functioning (items 1–5), Role Functioning (items 6 and 7), Dyspnoea (item 8), Pain (item 9 and 19), and Social Functioning (items 26 and 27) loaded onto factor 1 (Physical Health) and the domains Cognitive Functioning (item 20), and Emotional Functioning (items 21–24) loaded onto factor 2 (Mental Health). The sample size for the CFA, using the Physical/Mental Health model discussed above, was 1036; 124 patients had missing data for at least one of the 28 items. The comparative fit index was 0.97, the Tucker–Lewis fit index was 0.95, and the root mean squared error of approximation was 0.05 (90% CI 0.048–0.057; *P‐*close = 0.20). In addition, the coefficient of determination was 0.95 (interpreted as 95% of the variance in the observed variables being explained by the model) and the standardized root mean squared residual was 0.034.

The differences between Physical/Mental Health, stratified by the sociodemographic characteristics, are shown in Table 4. Again, there were differences among the various categories within each stratum, amongst, differences between the 1) most invasive tumour stage pT4 (*n* = 17) and non‐invasive tumour stage pT1 or below (*n* = 890), 2) the most comorbidities (>5) and the non‐comorbidity, were both larger than 10 points. Apart from tumour stage, the biggest difference was observed in the age strata, where patients aged >75 years and those aged <65 years differed by 8 points for Physical Health. The Physical and Mental health domains had a good correlation (*r* = 0.82).

**Table 4:** Physical and mental health scores across the different sociodemographic variables.

Demographic	Physical health		Mental health	
	Mean	sd	Mean	sd
Gender				
Male	80***	20	81*	17
Female	75	22	78	19
Age				
≤65 years (1)	83***	20	81*	19
66–75 years (2)	80	20	82	16
>75 years (3)	75	21	79	18
Marital status				
With partner	75***	23	79	20
Without partner	81	21	81	17
Smoking status				
Current	78*	23	79	21
Former	78	21	80	18
Never	82	20	83	17
Tumour stage				
pTis	82***	21	85**	13
pTa	81	19	82	16
pT1	79	21	81	18
pT2	75	23	78	18
pT3	73	–	87	–
pT4	69	32	71	25
NMIBC	80***	20	81*	17
MIBC	74	23	78	19
Comorbidity				
0	94***	14	87***	17
1	93	13	86	15
2	88	17	86	16
3	82	19	80	17
4	83	20	81	18
5	79	22	80	17
>5	70	25	73	23

MIBC, carcinoma invading bladder muscle; NMIBC, non‐muscle‐invasive bladder cancer. *P < 0.05; **P < 0.01; ***P < 0.001.

## Discussion

To our knowledge, this study is the first to investigate HRQoL in patients with NMIBC and those with MIBC at this specific time point: around the time of diagnosis. Interestingly, the HRQoL of both the NMIBC and MIBC groups was relatively high: none of the mean values for each domain fell below 60, and patients in both groups even scored >90 in a number of domains. In addition, we identified that reduced HRQoL was common in women and in patients with the features of aging and advanced tumour stage. Furthermore, patients with bladder cancer who had more comorbid conditions were much more likely to report poor HRQoL.

It is important to recognize that people with bladder cancer may have a different baseline HRQoL compared with the general population, as the disease may have already had an impact at or around the time of diagnosis. No reference data are available for the general population in the UK; however, the mean scores of HRQoL (QLQ‐C30) for the present study population were lower than data for the general populations of Germany, Norway, Sweden and Slovenia 30, 31, 32, 33, but higher than the general population of the USA 34. It is therefore, possible that HRQoL reference values for patients with bladder cancer depend on cultural factors; for example, Scandinavian populations have a high standard of living 35. This could lead to a greater difference in HRQoL among patients with bladder cancer from different countries.

Although it is more likely that cancer treatment (i.e. surgery, chemotherapy, radiation) will have an effect on HRQoL, an immediate change to an individual's HRQoL at diagnosis may depend on the type of cancer. Compared with people in the UK diagnosed with several other cancer types, including breast 36, lung 37, 38, colorectal 39, head and neck 40, malignant mesothelioma 41, endometrial cancer 42, oral and oropharyngeal cancer 43, the patients with bladder cancer in the present study had higher HRQoL scores for all domains, which might be attributable to the poorer prognoses for these other cancer types.

No clinically significant differences (>10 points) in HRQoL domains were found between patients with NMIBC and those with MIBC, except for Role Functioning. This might be attributable to the unique time point at which the questionnaire was administered, namely, before histopathological confirmation of diagnosis. Previous studies have found significantly worse HRQoL values between 3 and 18 months after diagnosis 44, 45, 46. However, in the present study it was apparent that the disease had already affected patients such that a difference between patients with NMIBC and MIBC could be identified in the Role Functioning domain (indicating that patients with MIBC feel more restricted in fulfilling their role in society) even at this early time point in the patient pathway. Nevertheless, patients with MIBC did not report higher pain levels or more restrictive Physical Functioning, possibly in contrast to what might be expected 47.

As demonstrated by multivariate analyses, age, gender, stage and comorbidity significantly influenced HRQoL. Overall, increased age, stage and comorbidity quantities were related to worse HRQoL. The tendency for women with bladder cancer to have a lower HRQoL than men with bladder cancer is consistent with some study findings in the general population 30, 31, 48, 49 and in other cancer types 50, 51, and also with earlier population studies showing that, on average, women reported a higher proportion of health problems 52.

It was not surprising that age was observed to be an important factor for defining HRQoL. Older participants experienced a deterioration for most of the functional scales, except for Emotional Functioning which was slightly increased in older people, and Social Functioning which was found to be unrelated to age. These findings are in line with earlier studies in general populations from other European countries 30, 31, 32, 33.

Comorbidity may in itself influence HRQoL because of the unique physical impact of the specific health condition and may thus play an important role when assessing HRQoL at the time of bladder cancer diagnosis. Indeed, we observed that comorbidity related to a decreased HRQoL, indicating that the presence of comorbidity before bladder cancer diagnosis may already have caused decrements in HRQoL. Since a more advanced tumour stage might cause more fear of progression and death, it was not unexpected to observe that tumour stage negatively affected HRQoL. Previous studies in other cancer types (e.g. oropharyngeal cancer 53 and prostate cancer 54) confirmed this finding. Both tumour‐specific features and comorbidity should be evaluated in patients with bladder cancer for better understanding of HRQoL.

It should be noted, however, that not all of the statistical differences observed in our multivariate analysis met the definition of clinical impact (all β coefficients <10); women were the exception, having substantially worse scores in the Insomnia domain. Furthermore, the β coefficient for age was relatively small per 1‐year difference, further implying limited clinical impact. The biggest impact of age can be found in Physical Functioning (β = 0.56), although a 10‐year age difference would only alter a patient's score by 5.6 points (on a scale from 0 to 100).

The factor analyses showed similar results to those of Gundy et al. [24]: we detected a two‐factor model for the analyses of the QLQ‐C30, even though not all the same items could be retained. We tested this model with CFA, which resulted in a well‐fitting model. Interestingly, the items that did not load highly onto the factors were single‐item symptom domains such as, Insomnia, Appetite Loss, Constipation, Diarrhoea, and Financial Difficulties. This could suggest that they do not contribute as much to overall HRQoL in these patients. Having a two‐factor model indicating Physical and Mental Health would increase the usability of the HRQoL questionnaire QLQ‐C30.

The EORTC has published a comprehensive report containing QLQ‐C30 reference values for a wide variety of cancers 55, 56. Compared with these reference values for patients with cancer overall 57, BCPP participants had similar scores. EORTC reference scores for patients with prostate cancer in stage I–II were similar to patients with NMIBC in the BCPP (although patients with prostate cancer did seem to have better Physical Functioning) and scores for patients with MIBC in the BCPP had similar scores to patients with stage III–IV prostate cancer (except for Physical Functioning, which was worse in those with prostate cancer). Reference values for patients with lung cancer were worse than for patients with MIBC in the BCPP cohort, with the exception of Cognitive Functioning values, which were similar.

As no UK QLQ‐C30 data are available for patients with bladder cancer at or around the time of diagnosis, the aim of the present study was to provide the first such data. To assess the generalizability of the present study population and findings, we compared our study demographics with the West Midlands Cancer Intelligence Unit data. We found no major differences with regard to age and gender (data not shown). In addition, the distribution of patients with NMIBC (78%) and MIBC (22%) in the BCPP corresponds with that in the literature from the UK 58; therefore, the results of the present study could be used as a reference population for future research into the HRQoL of patients with bladder cancer at or around the time of diagnosis in the UK and beyond.

It is important to note that these data from the BCPP have been captured from patients presenting in a conventional manner, having been referred from primary care to secondary care for the further investigation of symptoms, predominantly in a haematuria clinic setting; however, up to 18.5% of patients with bladder cancer present as emergency cases 59, and it is likely that these patients will have different HRQoL than those presenting conventionally.

In conclusion, this is the largest NMIBC patient cohort that has participated in HRQoL research. The study captured the HRQoL at a unique time point, allowing the identification of a baseline HRQoL measure. Our findings suggest that patients’ HRQoL scores at or around the time of bladder cancer diagnosis are very similar to those of the general population of Germany and possibly the UK. Only the domain Role Functioning was found to be significantly better in patients with NMIBC than those with MIBC at this time point.

The HRQoL scores given in the present study are interesting from a clinical point of view. Comparisons with HRQoL scores at the time of diagnosis give a better insight into any changes in a patient's HRQoL attributable to the disease or treatment.

## Conflict of Interest

None declared.
